# 

**Published:** 1886-02

**Authors:** 


					﻿Vol. VIJ.
FEBRUARY, 1 886.
No. 2.
THE
immtniFram loner
> MOTTHLI i'OWKAL
DENTAL AND ORAL SCIENCE.
W. C. BARRETT, M. D., D. D. S.,
EDITOR.
The New York Dental Journal Association,
PUBLISHERS,
No. 35 West 46th Street, New York.
AND
No. J3O8 Franklin St., Buffalo, N.Y.
$2.50 Per Annum in Advance, .... Single Copies, 25 Cents.
Entered at the Post-Office, Buffalo, N. Y., as Second-Class matter.
				

